# Independent regulation of renin–angiotensin–aldosterone system in the kidney

**DOI:** 10.1007/s10157-018-1567-1

**Published:** 2018-03-29

**Authors:** Akira Nishiyama, Hiroyuki Kobori

**Affiliations:** 1 Department of Pharmacology, Faculty of Medicine, Kagawa University, 1750-1 Miki-cho, Kita-gun, Kagawa 761-0793, Japan; 2 Departments of Pharmacology and Nephrology, Faculty of Medicine, International University of Health and Welfare, Narita, Japan

**Keywords:** Renin–angiotensin–aldosterone system (RAAS), Angiotensin II (Ang II), Angiotensinogen, Kidney

## Abstract

Renin–angiotensin–aldosterone system (RAAS) plays important roles in regulating renal hemodynamics and functions, as well as in the pathophysiology of hypertension and renal disease. In the kidney, angiotensin II (Ang II) production is controlled by independent multiple mechanisms. Ang II is compartmentalized in the renal interstitial fluid with much higher concentrations than those existing in the circulation. Inappropriate activation of the intrarenal RAAS is an important contributor to the pathogenesis of hypertension and renal injury. It has been revealed that intrarenal Ang II levels are predominantly regulated by angiotensinogen and therefore, urinary angiotensinogen could be a biomarker for intrarenal Ang II generation. In addition, recent studies have demonstrated that aldosterone contributes to the progression of renal injury via direct actions on glomerular podocytes, mesangial cells, proximal tubular cells and tubulo-interstitial fibroblasts through the activation of locally expressed mineralocorticoid receptor. Thus, it now appears that intrarenal RAAS is independently regulated and its inappropriate activation contributes to the pathogenesis of the development of hypertension and renal disease. This short review article will focus on the independent regulation of the intrarenal RAAS with an emphasis on the specific role of angiotensinogen.

## Introduction

The renin–angiotensin–aldosterone system (RAAS) is a hormone system that regulates blood pressure and fluid/electrolyte balance [1]. Angiotensin II (Ang II) binds to Ang II type 1 (AT_1_) receptor on vascular smooth muscle cells and tubules, which causes vasoconstriction and sodium reabsorption, respectively, leading to elevating blood pressure [2]. Ang II also binds to AT_1_ receptor on the adrenal gland to stimulate aldosterone production, which increases sodium reabsorption through the activation of mineralocorticoid receptor (MR) at distal nephron [3].

It now appears that local activation of intrarenal RAAS plays an important role in the pathogenesis of hypertension and renal tissue injury [4]. A number of studies have shown that progression of proteinuria and renal tissue injury are associated with an activation of intrarenal RAAS [5–9]. It has also been shown that treatment with angiotensin converting enzyme (ACE) inhibitors and Ang II AT_1_ receptor blockers (ARBs) significantly decrease proteinuria in patients with CKD, independently of blood pressure changes [10]. We previously showed that activation of intrarenal RAAS preceded the onset of micro-albuminuria in type 2 diabetic rats [11]. Furthermore, early treatment with ARBs attenuated the progression of albuminuria and renal injury [6, 12]. These data suggest the specific contribution of intrarenal RAAS activation to the pathophysiology of proteinuria and renal injury.

During treatment with an ARB, accumulation of Ang II can theoretically compete with ARBs at the receptor binding site. On the other hand, increase in Ang II during ARB treatment allows stimulation of the AT_2_ receptor [4]. Activation of the AT_2_ receptor is associated with increased release of nitric oxide, guanylate cyclase, and tissue bradykinin [13]. In contrast to the AT_1_ receptor, the AT_2_ receptor has antigrowth properties and stimulates programmed cell death. Thus, the AT_2_ receptor seems to counterbalance the effects of the AT_1_ receptor [14].

In this review, we will briefly summarize our current understanding of independent regulation of the intrarenal RAAS with an emphasis on the specific role of angiotensinogen. The mechanisms responsible for aldosterone-induced renal injury have been reviewed previously [1, 15, 16] and will not be discussed in detail in this review.

## Regulation of circulating classical RAAS pathways (Fig. 1)

**Fig. 1:**
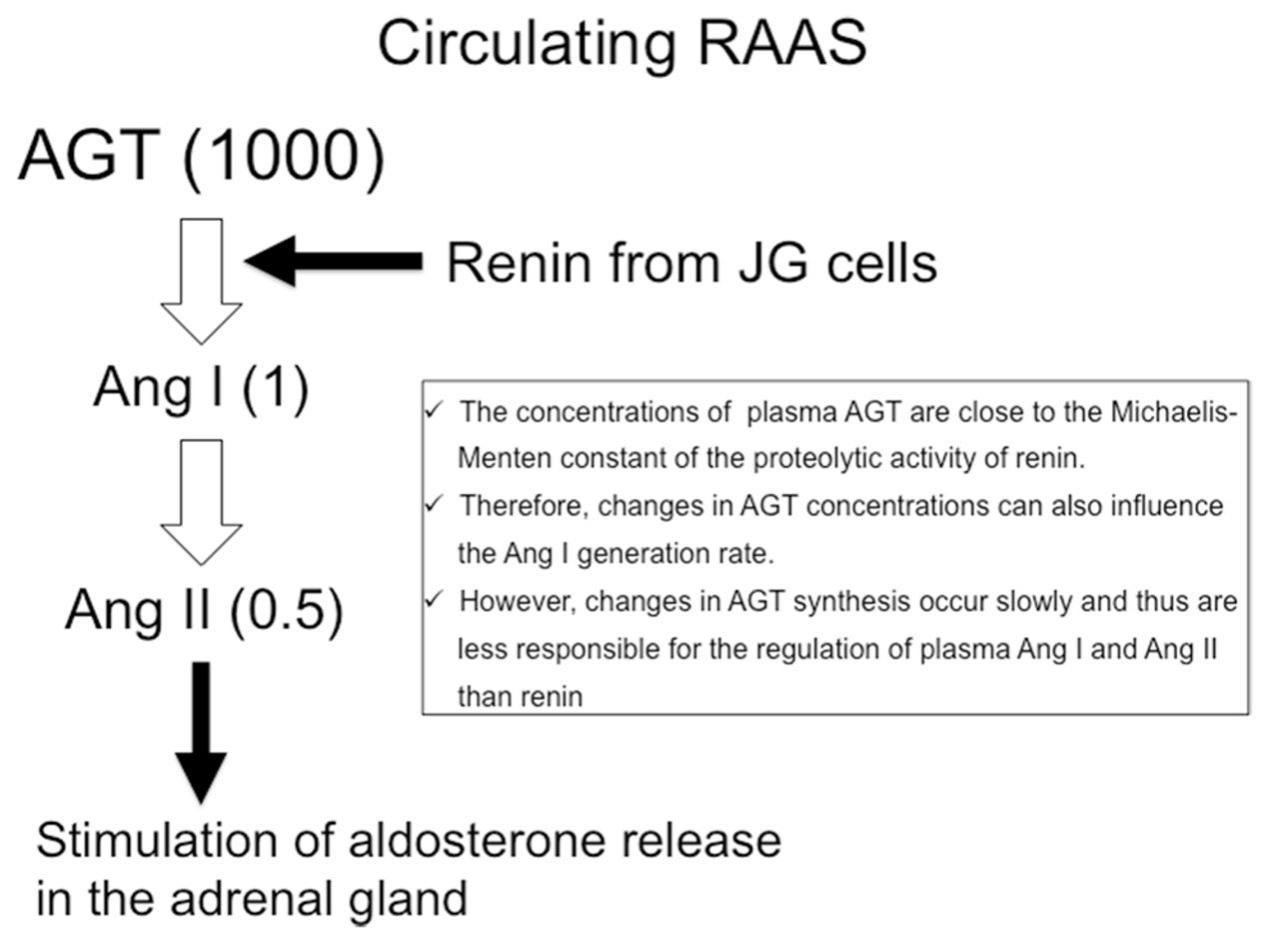
Brief scheme of circulating renin–angiotensin–aldosterone system (RAAS) regulation. *AGT* angiotensinogen, *Ang I* angiotensin I, *Ang II* angiotensin II, *JG cell* juxtaglomerular cell, *MR* mineralocorticoid receptor

Before discussing the regulation of intrarenal RAAS, the classical RAAS regulation in the plasma will be discussed [1, 4]. Angiotensinogen is the only known substrate for renin, which is the rate-limiting enzyme of the RAAS. Angiotensinogen is primarily formed by hepatic cells and constitutively secreted into the circulation [17, 18], whereas renin is released primarily from the juxtaglomerular cells of the kidney [4, 19] and cleaves angiotensinogen at the N terminus to form angiotensin I (Ang I) [20]. In plasma, angiotensinogen levels are much abundant, being more than 1000 times greater than the concentrations of Ang I and Ang II [7]. Because plasma levels of angiotensinogen are close to the Michaelis–Menten constant for renin, angiotensinogen levels can control plasma Ang I levels [17, 21]. Indeed, it has been shown that upregulation of angiotensinogen levels leads to elevated plasma Ang II levels [22, 23]. However, changes in angiotensinogen synthesis occur slowly, and thus are less responsible for the dynamic regulation of plasma Ang I [17, 24]. Therefore, it has been suggested that changes in plasma renin activity (PRA) play a predominant role in the determination of the rate of Ang I formation from the huge stores of circulating angiotensinogen in the plasma [1, 25]. Figure 1 shows the representative plasma angiotensinogen concentrations measured in anesthetized rats and expressed as nanomoles per liter, while the Ang I and Ang II concentrations are expressed as picomoles per liter. As shown in Fig. 1, the concentrations of Ang I and Ang II in the plasma seem to be small fractions of the available angiotensinogen, which supports the concept that renin is a critical factor to determine the Ang II generation in plasma [1, 18, 25]. Plasma Ang I can be easily converted to Ang II, due not only to the circulating soluble type of ACE, but also due to the widespread presence of ACE on endothelial cells of many vascular beds including the lung [2, 18]. Although other pathways for Ang II formation from Ang I have been identified [26], the circulating levels of Ang II reflect primarily the consequences of the renin and ACE enzymatic cascade on angiotensinogen and Ang I [27, 28]. Circulating Ang II binds to AT_1_ receptor on the adrenal gland to stimulate aldosterone production, which increases sodium reabsorption through the activation of MR at distal nephron [3].

## Regulation of local RAAS pathways in the kidney (Fig. 2)

**Fig. 2:**
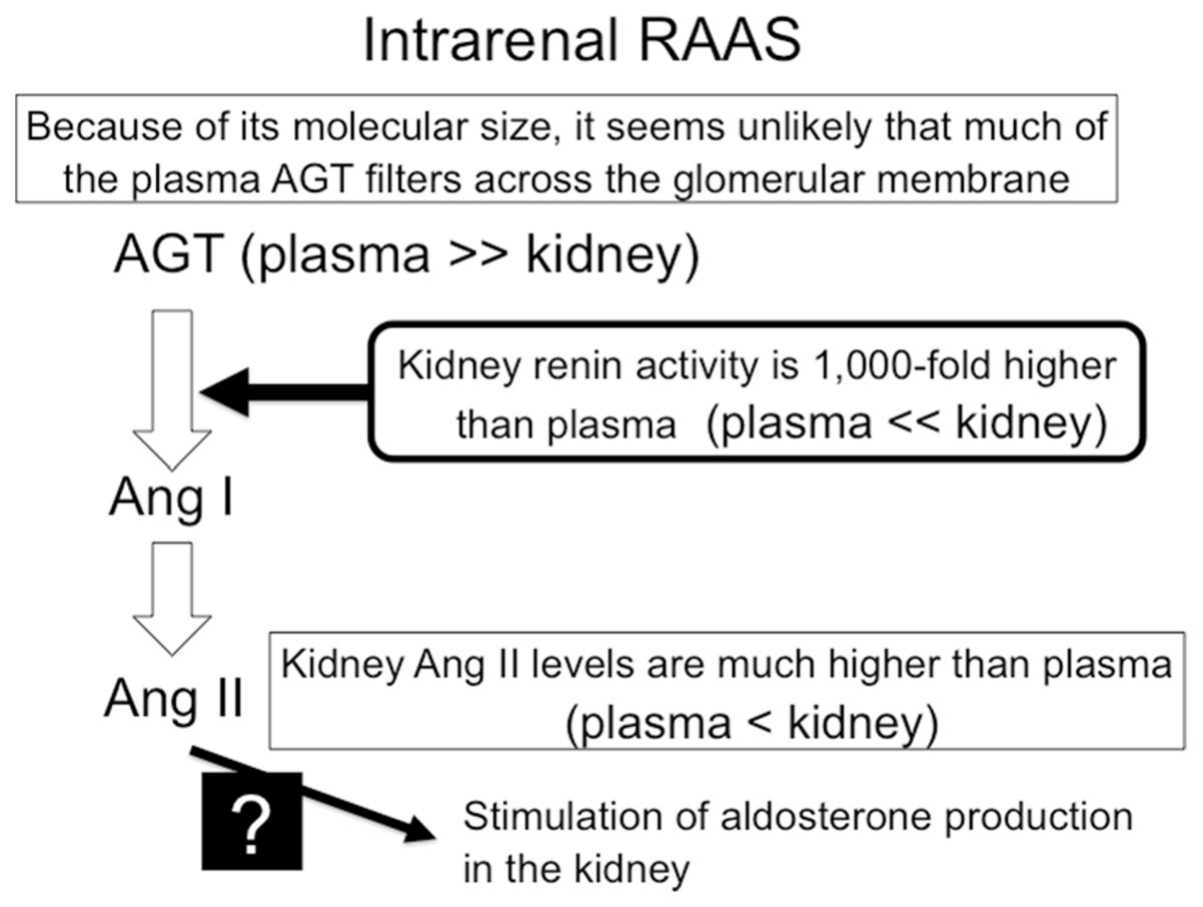
Brief scheme of intrarenal renin–angiotensin–aldosterone system (RAAS) regulation. *AGT* angiotensinogen, *Ang I* angiotensin I, *Ang II* angiotensin II

In the kidney, Ang II production is controlled by independent multiple mechanisms [4]. All of the components necessary to generate intrarenal Ang II are present along the nephron [2, 7]. Ang II concentrations in renal tissues are much greater than can be explained by the concentrations delivered by the arterial blood flow [4, 29]. Plasma angiotensinogen may not filter across the glomerular membrane because of its molecular size, but the kidneys also express angiotensinogen [5, 30]. However, angiotensinogen levels in renal tissues are much less as compared with those in plasma [4]. On the other hand, renin is secreted by the juxtaglomerular apparatus cells and delivered to the renal interstitium that provides a pathway for the local generation of Ang I in the kidney [19]. In particular, studies have suggested that renin activity in renal tissue is over 1000-fold higher than PRA (picomolar levels of Ang I/mL plasma/hour vs. nanomolar levels of Ang I/g tissue/h) [31]. Thus, abundant renin may easily cleave angiotensinogen to form Ang I. Furthermore, Ang I can also be easily converted into Ang II in the kidney [32, 33], because ACE is abundantly expressed in the proximal and distal tubules, the collecting ducts, and renal endothelial cells [34]. Collectively, unlike the role of renin in plasma, angiotensinogen is a critical factor to regulate Ang II production in the kidney. On the other hand, detail mechanism responsible for the Ang II-induced aldosterone production in the kidney has still not been clarified (Fig. 2).

Studies have indicated the compartmentalization and independent regulation of renal interstitial and tubular fluid Ang II. Ang II concentrations in the renal interstitial fluid are much higher than plasma levels [35]. Nishiyama et al. [36] showed that renal interstitial infusion of ACE inhibitors significantly decreased Ang II levels in renal interstitial fluid. These data indicate that Ang II is generated in the renal interstitial space. It has also been shown that Ang II concentrations in proximal tubular fluid are 100–200-fold higher than that in plasma [37, 38]. These results suggest that Ang II is also synthesized in the lumen of the proximal tubule, at least in part [39, 40].

In addition to intrarenal generation of Ang II, circulating Ang II is internalized in the kidney through the AT_1_ receptor [41]. Li et al. [42] showed that intrarenal trafficking/accumulation of Ang II into renal cortical tubular endosomes is enhanced during the development of Ang II-induced hypertension. Importantly, treatment with an ARB blocks an internalization of Ang II in the kidney.

## Specific role of angiotensinogen in the regulation of Ang II production in the kidney

In the kidney, angiotensinogen mRNA and protein have been mainly localized to proximal tubule cells [43, 44]. The angiotensinogen produced in proximal tubule cells seems to be secreted directly into the tubular lumen and renal interstitium in addition to producing its metabolites intracellularly [45]. Proximal tubule angiotensinogen concentrations in anesthetized rats have been reported to be in the range of 300–600 nmol/L, which greatly exceed the Ang I and Ang II levels in tubular fluid [7]. Transgenic mice that express human renin systemically and human angiotensinogen only in the kidney showed elevated intrarenal Ang II levels, while plasma Ang II levels were not changed. Interestingly, in these mice, endogenous mouse angiotensinogen expression was also augmented [23]. Thus, the selective stimulation of intrarenal production of Ang II from human angiotensinogen further stimulates endogenous intrarenal mouse angiotensinogen expression. Similarly, intrarenal angiotensinogen expression is augmented in Ang II-infused hypertensive rats [46, 47]. Chronic Ang II infusions also significantly increased the urinary excretion rate of angiotensinogen in a time- and dose-dependent manner that were associated with elevations in systolic blood pressure and kidney Ang II levels but not with plasma Ang II concentrations [30]. Furthermore, treatment with an ARB prevented the Ang II-induced augmentation of angiotensinogen expression in the kidney and urinary angiotensinogen [30]. These data suggest that angiotensinogen production in the kidney is positively stimulated by local Ang II through the activation of AT_1_ receptor. Further studies have shown that high glucose stimulates angiotensinogen gene expression in human proximal tubular cells [48, 49]. Furthermore, in renal tissues of type 2 diabetic rats [6, 11, 44, 50] and patients [51], gene expression of angiotensinogen was significantly increased in the kidney. Thus, it can be speculated that during the development of diabetes, high glucose initially increases intrarenal angiotensinogen levels, leading to generation of Ang II in the kidney. Then, inappropriate production of Ang II may further stimulate local expression of angiotensinogen and associated Ang II generation in the kidney. Such vicious cycle of intrarenal RAAS activation is suggested to be a critical factor for the progression of diabetic nephropathy [52, 53]. Studies have also shown that treatment with ARBs significantly decreases both angiotensinogen expression and Ang II levels in the kidney [6, 10, 12, 54, 55]. Thus, pharmacological renoprotective effects of ARBs could be partially explained by inhibiting the production of intrarenal angiotensinogen and Ang II. Although renal angiotensinogen is predominantly localized in the proximal tubules [44, 46, 47], weak expression is also detected in glomeruli. Since glomerular angiotensinogen is increased in damaged glomeruli [56–58], local RAAS activation in glomerulus may play a role in the pathophysiology of glomerular injury. Recently, Eriguchi et al. [56] have shown that angiotensinogen is generated in injured glomerular podocytes in nephrotic rats induced by puromycin. These data suggest the potential contribution of podocyte angiotensinogen generation in the progression of proteinuria.

In addition to two factors, Ang II and high glucose, other factors such as mitogen-activated protein kinases (MAPK), reactive oxygen species (ROS), and nuclear factor kappa-light-chain-enhancer of activated B cells (NFkB), were also reported to activate angiotensinogen expression. Zhang et al. [59] showed that angiotensinogen gene expression is stimulated via p38 kinase pathway in immortalized proximal tubular cells of rat kidney. Hsieh et al. [60] found that angiotensinogen gene expression is activated via ROS in a proximal tubular cell line. In addition, Kobori and Nishiyama [61] presented evidence in vivo that ROS stimulates angiotensinogen gene expression in kidneys of Dahl salt-sensitive rats challenged by a high salt diet. Finally, angiotensinogen gene expression is activated by NFkB p65 transcription factor in hepatocytes [62]. Possible linkage between MAPK activation and NFkB pathways has also been suggested [63, 64].

## Urinary angiotensinogen as a biomarker of intrarenal RAAS and renal injury

As mentioned before, plasma angiotensinogen may not easily filter across the glomerular membrane because of its molecular size. Ding et al. [65] generated kidney-specific human angiotensinogen overexpression mice and found abundant human angiotensinogen in the urine, but only slight traces in the systemic circulation. Kobori et al. [30] infused human angiotensinogen intravenously in rats; however, circulating human angiotensinogen was not detectable in the urine. Further studies with two-photon microscopy visualized glomerular dynamics in vivo and showed glomerular filtration of circulating human angiotensinogen is much less as compared with albumin in mice, suggesting limited glomerular permeability [66].

As angiotensinogen is a protein, one may think that in subjects with proteinuria, the increased urinary excretion of angiotensinogen is a non-specific consequence of the increased urinary excretion of plasma protein [67, 68]. However, urinary angiotensinogen was not augmented, although urinary protein is elevated in deoxycorticosterone acetate-treated rats, a model of RAAS-independent hypertension [30]. In addition, the urinary angiotensinogen/creatinine ratio in patients with minor glomerular abnormality (8.3 ± 3.7 μg/g Cr) was similar to that in healthy subjects (10.8 ± 3.4 μg/g Cr), even though these patients showed severe proteinuria [9]. In pre-albuminuric patients with type 1 diabetes, urinary angiotensinogen levels were already higher than in control subjects [69]. Zhuang et al. [70] have shown that elevated urinary angiotensinogen levels precede the onset of albuminuria in patients with type 2 diabetes. Similarly, urinary angiotensinogen and sodium excretions are significantly increased in normo-albuminuric children with diabetes [71]. These data suggest that enhanced urinary angiotensinogen levels in patients with proteinuria cannot be simply explained by a non-specific consequence of proteinuria.

However, circulating angiotensinogen may be filtered across the glomerular membrane under some pathophysiological conditions. Indeed, several clinical studies have shown that urinary angiotensinogen levels are significantly correlated with albuminuria in patients with hypertension [72] and CKD [67, 73, 74]. Matsusaka et al. [75, 76] have shown that in mice with severe podocyte injury and loss of macromolecular barrier function of the glomerular capillary wall, intrarenal Ang II generation is augmented by filtered angiotensinogen originated from the liver. Eriguchi et al. [56] have shown that during the progression of podocyte injury, filtered angiotensinogen is abundantly re-absorpted by proximal tubules, leading to reduction in proximal tubular angiotensinogen generation. It has also been suggested glomerular podocyte is an important source of urinary angiotensinogen in this pathological condition [9]. These data suggest that filtered circulating angiotensinogen can also be an origin and/or trigger of intrarenal Ang II generation in subjects with severe damage of glomerular filtration barrier, which may play an important role in the activation of intrarenal RAAS during the progression of CKD.

A growing body of evidence has shown that urinary angiotensinogen is a specific biomarker for the status of the intrarenal RAAS, hypertension and renal disease. Kobori et al. [30, 77] have conducted animal experiments and shown that increases in urinary angiotensinogen are associated with augmentation of renal angiotensinogen expression and Ang II levels in the kidney. In patients with minor glomerular abnormality and IgA nephropathy, urinary angiotensinogen/creatinine ratio is highly correlated with gene expression of angiotensinogen in renal biopsy tissues [9]. These data have indicated that urinary angiotensinogen is an useful marker for predicting the levels of angiotensinogen in the kidney of these patients. A sandwich enzyme-linked immunosorbent assay (ELISA) for human angiotensinogen was developed by Katsurada et al. [78] and it is now commercially available, which has made it easy to measure a large quantity of specimens over time. It has also been shown that usual preservation conditions do not affect the measured values of urinary angiotensinogen [79]. Furthermore, urinary angiotensinogen excretion has not a circadian rhythm [80]. Thus, investigations of urinary angiotensinogen have been widely spread in the world and a growing body of clinical evidence has indicated that augmented urinary angiotensinogen levels are correlated with clinical parameters in patients with hypertension [71, 72, 81] and CKD [55, 73, 82–84].

Kobori et al. [81] showed that urinary angiotensinogen was significantly correlated with blood pressure in hypertensive patients who were not treated with any anti-hypertensive agents. They also found that this correlation was high in Black men, suggesting the possible contribution of urinary angiotensinogen to salt-dependent hypertension. Interestingly, Kobori et al. [85] have also shown that both angiotensinogen expression in renal tissues and urinary angiotensinogen were markedly augmented in salt-treated Dahl salt-sensitive hypertensive rats. Konishi et al. [55] have shown that sodium sensitive index for blood pressure is highly correlated with urinary angiotensinogen in IgA patients with nephropathy who show sodium-dependent blood pressure elevation. Similarly, Zou et al. [86] have shown that urinary angiotensinogen excretion is higher with greater urinary sodium excretion, and is associated with both clinic and ambulatory blood pressure. Further studies have shown that an increase in urinary angiotensinogen is significantly correlated with urinary sodium and precedes hypertension in normo-albuminuric children with type 1 diabetes [71]. These data suggest that urinary angiotensinogen is a useful biomarker to identify sodium-dependent hypertension. Sawaguchi et al. [84] showed that urinary angiotensinogen was highly correlated with incidence of cardiovascular complications in patients with type 2 diabetic nephropathy. Recent studies have also shown that urinary angiotensinogen is significantly correlated with left ventricular mass index and intima-media thickness in hypertensive kidney transplant patients [87]. We have also shown that both urinary angiotensinogen and intrarenal angiotensinogen levels are significantly augmented in rats with aortic regurgitation [88], suggesting the potential role of intrarenal angiotensinogen in the pathophysiology of cardio-renal syndrome.

**Fig. 3:**
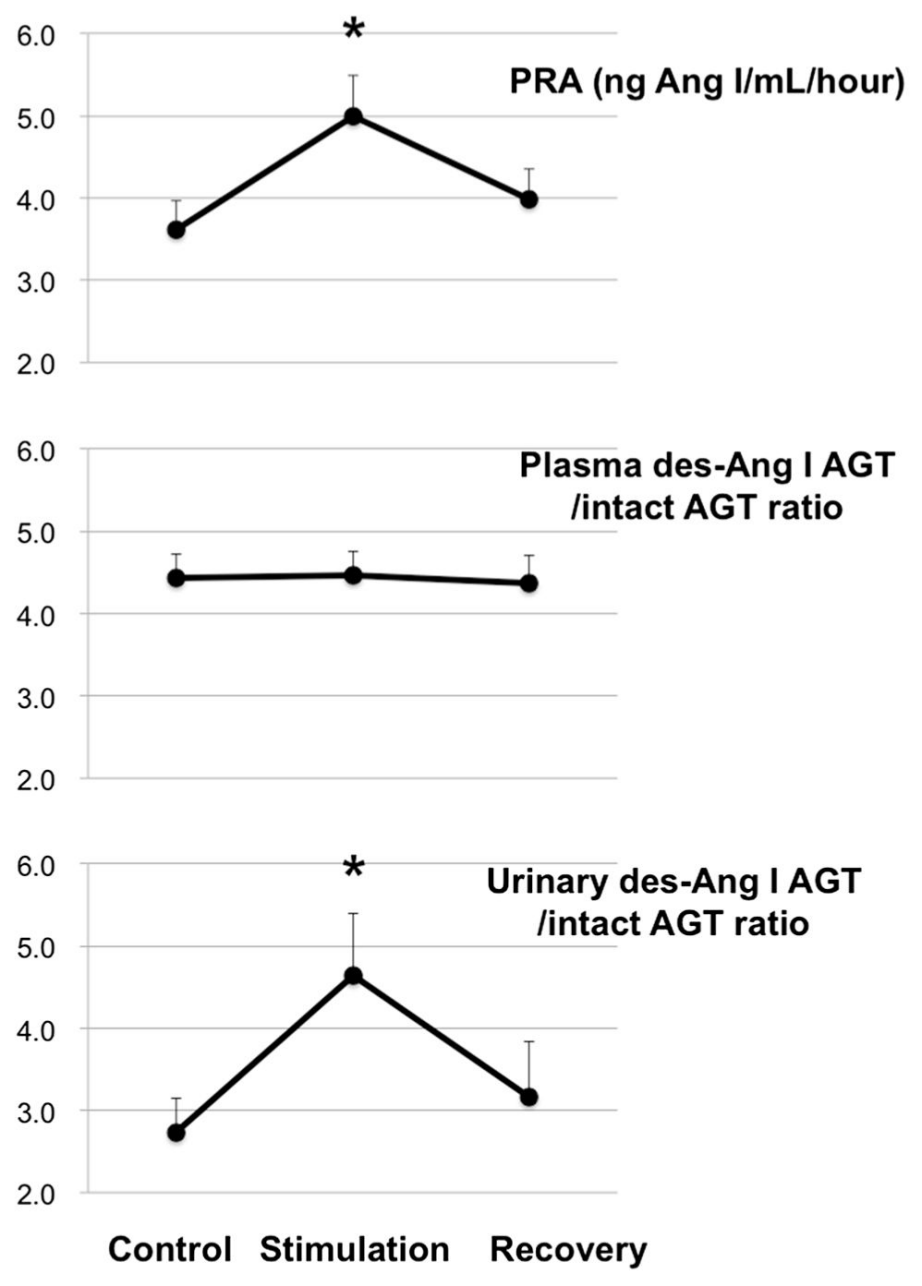
Effects of electrically stimulation of renal sympathetic nerve at 1 Hz (5 V, 1 msec) for 20 min on PRA, plasma des-Ang I-AGT/intact AGT ratio, and urinary des-Ang I-AGT/intact AGT ratio in anesthetized rats (*n* = 12–15). *PRA* plasma renin activity, *AGT* angiotensinogen, *Ang I* angiotensin I. *P < 0.05 vs. control

Several clinical studies have shown that urinary angiotensinogen is significantly increased in patients with CKD including IgA nephropathy [55], diabetic nephropathy [84, 87, 89], polycystic kidneys [90, 91], focal segmental glomerulosclerosis [56]. In these CKD patients, urinary angiotensinogen is positively correlated with urinary protein or albumin levels, while it is negatively correlated with estimated glomerular filtration rate. Recent studies have also indicated that urinary angiotensinogen is a prognostic biomarker for acute kidney injury [92, 93] and renal scarring [94]. It should be important, however, to note that the antibodies used in commercially available angiotensinogen ELISA assay kits recognize both intact angiotensinogen and des-Ang I angiotensinogen [78]. Angiotensinogen can be schematically considered to consist of a combination of an Ang I function, located at the N-terminal end, and the presence of a serpin (serine protease inhibitor) structure at the opposite end. Thus, further studies should be needed using a new ELISA kit for intact angiotensinogen. Kobori and Nishiyama have recently proposed that the ratio between des-Ang I angiotensinogen and intact angiotensinogen could be a stable marker for renin activity (A patent, PCT/JP2014/078751, was filed). Since plasma angiotensinogen levels are much abundant, we speculate that acute changes in renin activation may not reflect the des-Ang I angiotensinogen/intact angiotensinogen ratio in the plasma. To test this idea, we electrically stimulated renal sympathetic nerve at 1 Hz (5 V, 1 msec) for 20 min in anesthetized rats and collected plasma and urinary samples. All experimental procedures were carried out according to the guidelines for care and use of animals established by Kagawa University (Kagawa, Japan). Our preliminary data showed that activation of renal sympathetic nerve significantly increased PRA, but did not change plasma des-Ang I angiotensinogen/intact angiotensinogen ratio (Fig. 3). Interestingly, urinary des-Ang I angiotensinogen/intact angiotensinogen ratio was soon increased by renal sympathetic nerve stimulation. These data suggest that urinary des-Ang I angiotensinogen/intact angiotensinogen ratio is a potential biomarker for the activity of renin in the kidney.

Des-Ang I angiotensinogen, which accounts for more than 97% of the molecule, apparently has no function. Several serpins (antithrombin, maspin, pigment epithelial-derived factor, and kallistatin) have been recently shown to exert an anti-angiogenic activity, suggesting a common mechanism of endothelial cell proliferation and migration. Angiotensinogen and its renin-cleaved product, des-Ang I angiotensinogen, are also angiogenesis inhibitors, both in vitro and in vivo at concentrations within the range of those observed in plasma. This property most likely results from the structure analogy of angiotensinogen with serpins. The pathologic relevance of this new function is still not known, but angiotensinogen produced by glial cells may play a role in the stabilization of the blood–brain barrier. These new data must be considered in light of the overall action of the renin–angiotensin system in angiogenesis [95].

## Conclusions

Intrarenal RAAS is independently regulated and its inappropriate activation contributes to the pathogenesis of the development of hypertension and renal disease. This brief review discussed the specific role of angiotensinogen in the regulation of intrarenal RAAS activity. Locally expressed angiotensinogen is a major contributor to control intrarenal Ang II levels, but filtered circulating angiotensinogen can also be an origin of intrarenal Ang II generation if glomerular filtration barrier is severely damaged. In any case, urinary angiotensinogen is a useful biomarker for identifying the status of the intrarenal RAAS, hypertension and renal disease.
